# Case series of intradural disc in recurrence of lumbar disc prolapse

**DOI:** 10.1093/jscr/rjaa611

**Published:** 2021-02-27

**Authors:** Bashar Alhani, Satish Nagappa, Charles Baird, Rajesh Botchu, Simon Hughes, Jewalant Mehta, Faizul Hassan

**Affiliations:** Spinal Surgery, The Royal Orthopaedic Hospital Birmingham, Birmingham B31 2AP, UK; Spinal Surgery, The Royal Orthopaedic Hospital Birmingham, Birmingham B31 2AP, UK; Spinal Surgery, The Royal Orthopaedic Hospital Birmingham, Birmingham B31 2AP, UK; MSK Radiology, The Royal Orthopaedic Hospital Birmingham, Birmingham B31 2AP, UK; Spinal Surgery, The Royal Orthopaedic Hospital Birmingham, Birmingham B31 2AP, UK; Spinal Surgery, The Royal Orthopaedic Hospital Birmingham, Birmingham B31 2AP, UK; Spinal Surgery, The Royal Orthopaedic Hospital Birmingham, Birmingham B31 2AP, UK

## Abstract

Intradural disc herniation is a rare entity reported at 0.04–1.1% that occurs most commonly in the lumbar spine particularly at L4–L5 region.

There is a paucity of literature due to the rarity of this condition. Intradural disc herniations must be considered in the differential diagnosis of prolapsed intervertebral disc disease especially with recent worsening of symptoms and mismatch of unenhanced magnetic resonance induction (MRI) findings. The confirmation is made with intraoperative findings.

An intradural disc herniation is most often diagnosed intraoperatively. Contrast enhanced MRI scan is mandatory for pre-operative diagnosis.

We report on two cases presenting to our unit in the form of recurrent intradural disc disease following previous lumbar surgery occurring within 3 months of the index procedure in both cases.

## INTRODUCTION

Intradural disc herniation is a rare entity reported at 0.04–1.1% by Sharma *et al*. that occurs most commonly in the lumbar spine particularly at L4–L5 region [1]. Lechowski *et al*. presented 11 cases of intradural lumbar disc herniations from 2030 (<0.05%) patients treated surgically for intervertebral disc prolaps whereas Schisano *et al*. reported nine cases of intradural herniations (1.51%) in 593 cases of ruptured lumbar disc that underwent surgery from 1980 to 1992 [6]. All authors concluded that the frequency of intradural disc rupture is very low [1, 6, 7].

We report on two cases presenting to our unit in the form of recurrent intradural disc disease following previous lumbar surgery occurring within 3 months of the index procedure in both cases.

## CASE 1

**Figure 1:**
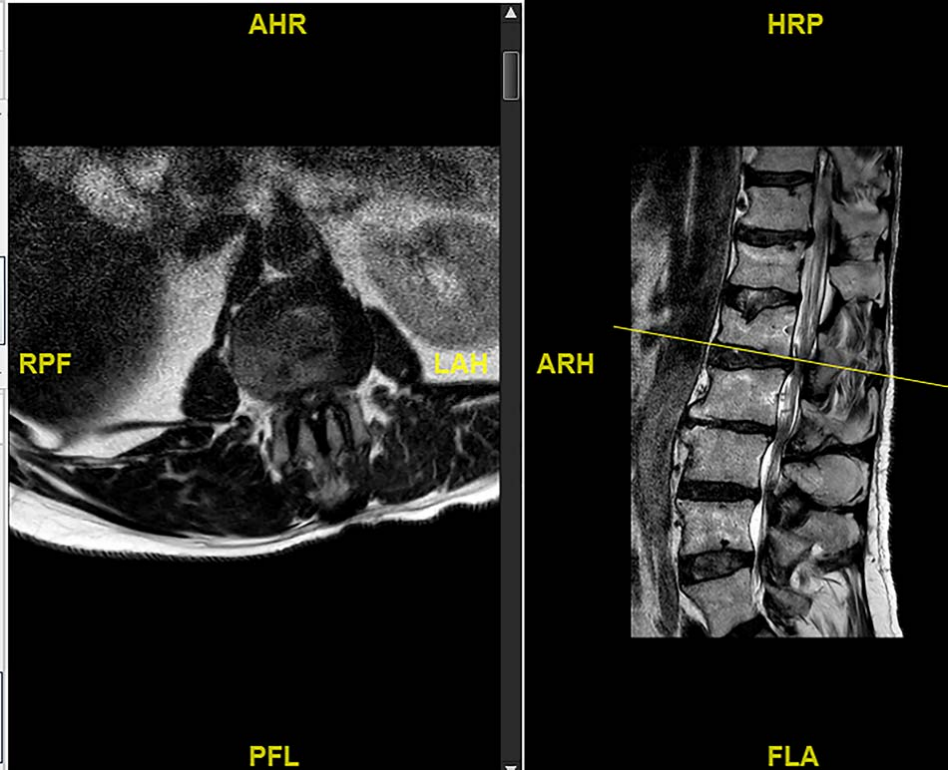
Initial MRI images prior to index procedure revealing stenosis at L1/2 and L2/3.

**Figure 2:**
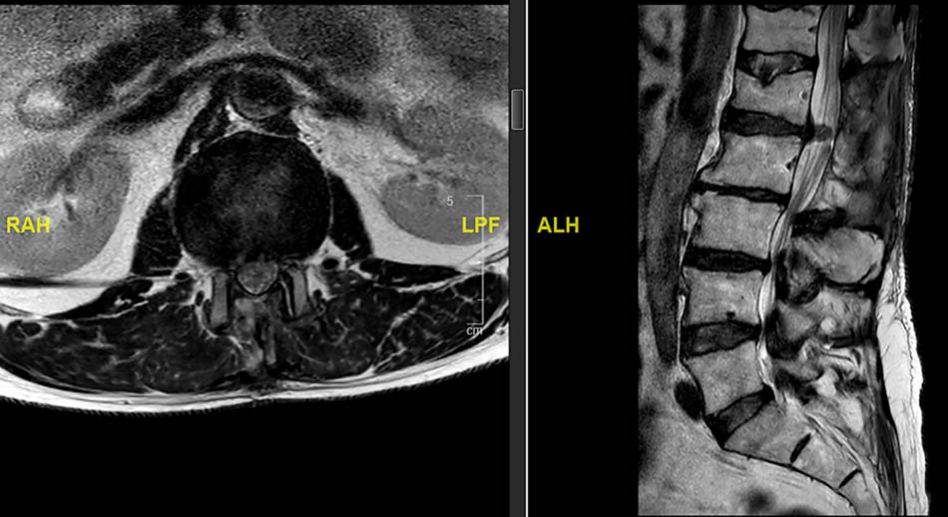
MRI image revealing a recurrence of a disc prolapse, suspected to be an intradural disc at L1/2, confirmed intraoperatively.

**Figure 3:**
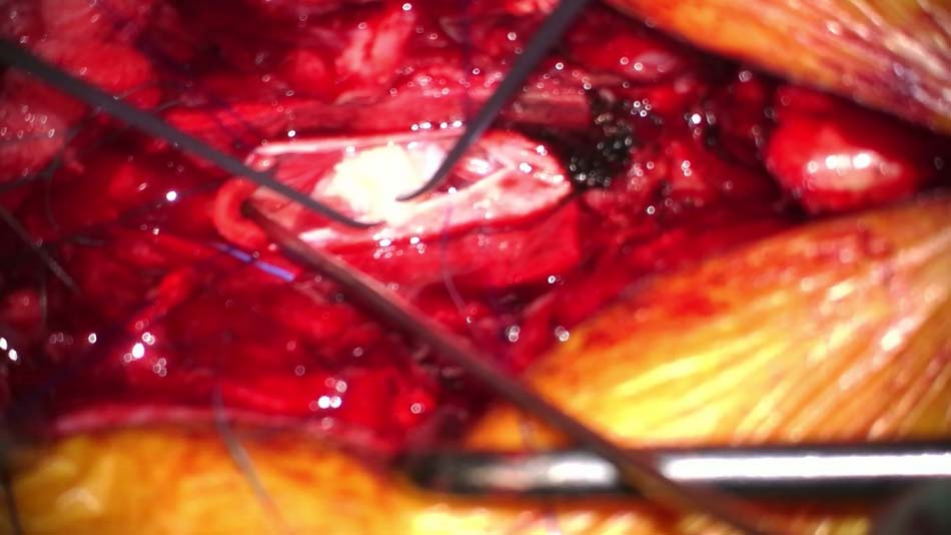
Intraoperative picture showing durotomy and intradural disc being delivered at L1/2.

A 56-year-old male patient lumber decompression in a local neurosurgical centre for severe canal stenosis at L1/2 and L2/3 (Fig. 1) level after presenting with bilateral leg weakness and perianal numbness. Three months later he presented with recurrence of right leg pain radiating into the calf and worsening back pain as well as distal lower limbs weakness bilaterally with no sphincter problem. Following an magnetic resonance induction (MRI), which revealed a disc prolapse at L1/2 (Fig. 2), the patient was referred to our Centre. The images were reviewed by a consultant radiologist and as a possibly of intradural herniated disc disease was described. Intraoperatively no extradural disc prolapse was found, so midline durotomy was performed. Intradural disc herniation (IDH) was identified, which was communicating with the disc space through a midline defect in anterior dura (Fig. 3). This was removed extracted under the microscope. Post-operatively, he had a CSF leak needing re-suturing of dura. The patient did make a good post-operative recovery with no major sensory or motor deficits or sphincter disturbance.

## CASE 2

A 54-year-old male with known case of mild disc prolapse at L4/5 and L5/S1 level presented with features of cauda equina syndrome (CES).

**Figure 4:**
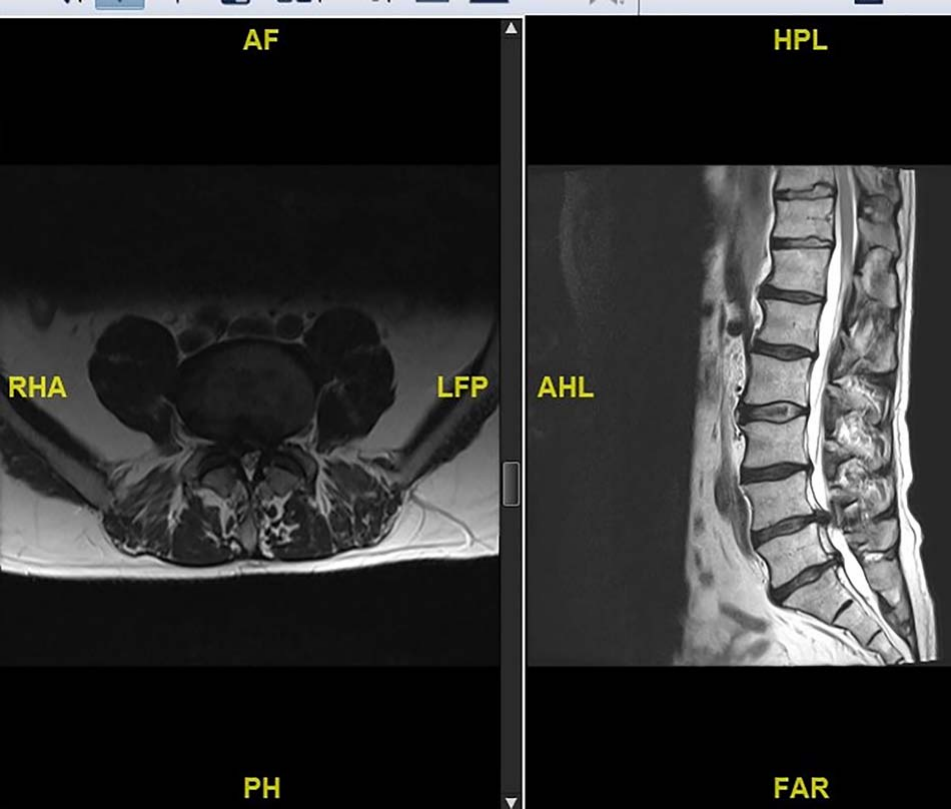
Preoperative MRI scan showing L4/5 disc prolapse.

The updated MRI scan showed large disc prolapse at L4/5 level (Fig. 4) and the patient underwent emergency laminectomy/discectomy at L4/5. Post-operatively the patient’s pain improved however there still a retained loss of perineal sensation to pin-prick, a urinary catheter tug awareness was present and a good lower limb power was documented. The patient was discharged on the third post-operative day having been successfully mobilized and decatheterized with spontanous micturition.

**Figure 5:**
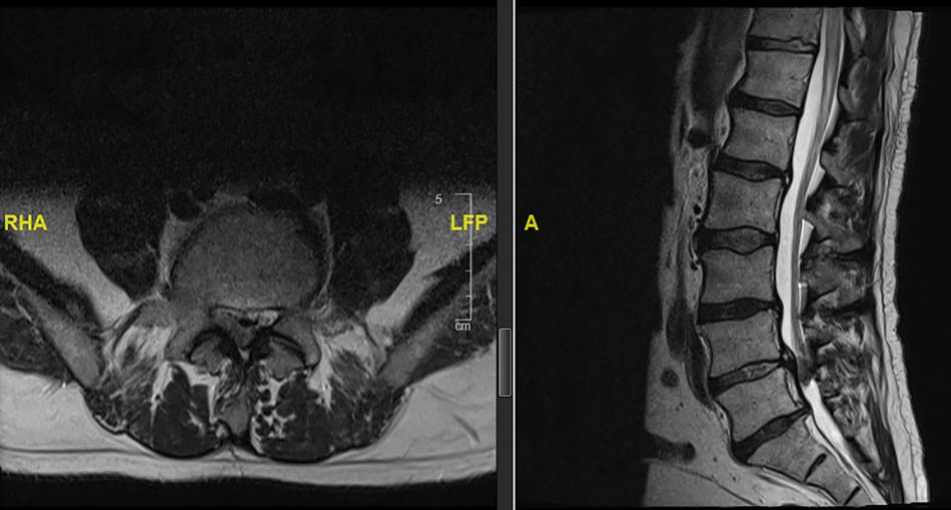
MRI scan showing an intradural disc at L4/5.

The patient represented just over a month following the surgery to report about new onset weakness in the plantar flexion bilaterally, urinary incontinence, persistence of perineal sensation loss and new erection problems. There was no report of any pain and ambulation was still at ease. An MRI scan was organized a few days later (Fig. 5). The finding was deemed to be an IDH. Therefore, revision surgery followed the next day, with a durotomy and extraction of intradural disc fragments. As the patient had sphincter disturbance with neurogenic bladder and bowel difficulties, a referral to the regional spinal injury unit for aftercare as well as to the sexual dysfunction clinic for erection problems were organised.

At review 2 months after the revision procedure, the patient still reported of improvement in the urinary function with fewer incontinence incidents, however was still in need of frequent aperients to regulate bowel movement. Lack of perineal sensation and sexual dysfunction remained.

## DISCUSSION

Intradural discal lesions are a rare entity. There is a paucity of literature due the rarity of this condition, although it has been randomly reported in literature [4]. Even further none of the reviewed literature has mentioned bilateral leg symptom as a feature of clinical presentation [3–7]. IDHs must be considered in the differential diagnosis of prolapsed intervertebral disc disease especially with recent worsening of symptoms and mismatch of unenhanced MRI findings. The confirmation is made through the intraoperative findings [10].

Yildizhan *et al*. analysed the relationship between the ventral dura and posterior longitudinal ligament in the cervical, thoracic, lumbar and sacral regions. The authors reported that the ventral dura was most frequently and firmly attached to the posterior longitudinal ligament at the L4/L5 level and that these adhesions may be congenital. In adult cadavers the dorsal dura was found to be thicker than the ventral dura in the lumbar and lower cervical interspaces [9].

The preoperative diagnosis of this condition should be suspected when there is a history of significant chronic low-back pain followed by an acute episode of CES and presence of a complete or nearly complete block on myelography [8]. In these cases, adhesions of dura mater with ligaments were always found [9]. The penetration of an extruded disc through the dura mater has been explained as being due to adhesions that attach the Dural sac to the anterior wall of the spinal canal.

Although extradural disc prolapse is the usual finding, clinically however, intradural disc disease tend to have more pronounced clinical features as we have experienced. Diagnosing intradural disc does pose a challenge as acknowledged from previous publications [1–7].

‘Y sign’ in ventral dura due to splitting of ventral dura and arachnoid mater by disc material was acknowledged to be a good diagnostic sign to suspect intradural extra-arachnoid disc. The presence of hypointense structure inside the dura with no continuity with the adjacent intervertebral disc on MRI was highly suggestive of an intradural disc [1].

Our reported cases clearly underline the importance of follow-up and work-up of patients who failed to improve after disc surgery. Both cases shared a bilateral leg symptom of pronounced dimension. This leads to conclusion that intradural breach by the disc fragments evokes a meningitic like reaction from our observation of the two patients reported in this series. This clinically could differentiate it from the ‘by far more common’ extradural disc disease, where unilateral sciatica is the lead symptom. Cauda equina features are the red flags, therefore a low threshold for early re-imaging is essential with the purpose of maximising neurologic outcome or minimising neurologic injury.
